# Molecular dynamics simulation study on macroscopic mechanical deterioration and microscopic hydration mechanism of mudstone

**DOI:** 10.1371/journal.pone.0356473

**Published:** 2026-08-19

**Authors:** Lidong Hou, Shaojie Li, Jinsong Bai, Changhao Wang, Yunzhao Liu, Ziyang Zhu

**Affiliations:** 1 National Key Laboratory of Continental Shale Oil, Northeast Petroleum University, Daqing, Hei Longjiang, China; 2 Heli(Tianjin) Robotechs Technology Co., Ltd., Tianjin, China; 3 Daqing Oilfield, China National Petroleum Corporation, Daqing, Hei Longjiang, China; Northeastern University, CHINA

## Abstract

Aiming at the common problem of borehole instability caused by mineral hydration in water-sensitive mudstone formations during petroleum drilling, this paper conducts a multi-scale study by integrating X-ray diffraction, triaxial mechanical experiments, and molecular dynamics simulation methods. It systematically analyzes the mineral composition of mudstone, the evolution law of its mechanical properties after hydration, and the microscopic mechanism of hydration. The results indicate that: ① The montmorillonite content in the mudstone dominates its strong water sensitivity, leading to a sharp deterioration in mechanical properties after immersion, with compressive strength decreasing by over 55%; ② Molecular dynamics simulation reveals that montmorillonite hydration is a staged process: water molecules intrude into the interlayer to sequentially form single-layer, double-layer, and triple-layer water film structures, with the interlayer spacing expanding nonlinearly. Na⁺ migrates accordingly and distributes alternately with water molecules. The diffusion coefficient of water molecules is significantly higher than that of Na ⁺ . These factors collectively cause a continuous decline in interlayer structural stability; ③ This microscopic process, from “staged formation of water films” to “gradual structural destabilization,” directly explains the macroscopic phenomenon of cores exhibiting progressive failure characterized by “initial spalling followed by fragmentation” with increasing immersion time. This study establishes a complete evolution path of mudstone hydration damage from the atomic scale to the core scale, providing a key theoretical basis for controlling borehole stability by inhibiting interlayer hydration of montmorillonite.

## 1. Introduction

Water-sensitive mudstone formations frequently encountered during drilling operations are prone to hydration expansion and mechanical property decay upon invasion by drilling fluid due to their internal clay minerals. This often leads to borehole instability issues such as wellbore collapse, diameter reduction, and cavings, severely affecting drilling efficiency and threatening downhole safety [1–5]. Therefore, in-depth research on the hydration mechanism of mudstone and its impact on borehole stability is of great significance for optimizing drilling technology and preventing engineering risks [6–9].

In recent years, domestic and international scholars have conducted extensive research on mudstone hydration [10–12]. Xiao et al.[13] experimentally analyzed the rock mechanical properties and basic physical characteristics of different formations, investigated the mechanism of drilling fluid hydration effects on rock mechanical properties and micro-pore structure, and subsequently improved inhibitors to reduce formation damage caused by drilling fluid. Fan et al.[14] conducted macro-measurement experiments on mudstone formations, analyzed the mineral composition and bedding structure characteristics of rock samples, determined the relationship between different water contents and weak plane strength, and established a formation collapse pressure calculation model. Jin et al.[15] reviewed research progress on the macroscopic mechanical deterioration effect of mudstone hydration and quantitative analysis of borehole stability. Regarding molecular dynamics studies on mudstone hydration, Xu et al.[16] used molecular dynamics simulation to study the variation patterns of sodium vermiculite (Na-VMT) with increasing water content and its swelling characteristics under different hydration states. Li et al.[17] used MD methods to study, from a microscopic perspective, changes in interlayer spacing, migration patterns of substances between layers, ion hydration parameters, and mechanical parameters of montmorillonite with different hydration degrees under varying temperature and pressure conditions. Liu et al.[18] elucidated the effect of cation radius on montmorillonite hydration by combining Static Multiple Light Scattering (SMLS), Quartz Crystal Microbalance with Dissipation Monitoring (QCM-D), and Molecular Dynamics (MD) Simulation. Zhou et al.[19] used molecular dynamics methods to simulate the effect of different temperatures and added inorganic salts on the hydration layer structure of rock walls, explaining the mechanism of temperature and inorganic salts inhibiting water molecules from a microscopic perspective.

Despite these efforts, the intrinsic link between the atomistic structural evolution of clay minerals and the consequent macroscopic wellbore instability is still poorly understood. In particular, previous molecular dynamics studies have rarely established a direct cross‑scale mapping between specific element‑substituted montmorillonite models and the macroscopic phenomenon of progressive core failure observed in immersion experiments. This study fills this gap by systematically correlating the staged interlayer hydration process with the time‑dependent mechanical deterioration of mudstone. This paper systematically studies the microscopic hydration mechanism of mudstone under the action of drilling fluid and the evolution law of its macroscopic mechanical properties by comprehensively applying methods combining mineral composition analysis, rock mechanics experiments, and molecular dynamics simulation. It aims to reveal the intrinsic relationship between interlayer structure evolution, ion migration, and macroscopic strength deterioration during mudstone hydration, providing a theoretical basis and technical support for optimizing drilling fluid systems and controlling borehole stability in mudstone formations.

## 2. Structural characteristics and physico-mechanical response of mudstone in a collapse-prone formation

Extensive development of water-sensitive mudstone intervals is observed in the Formation of Block S. These mudstones exhibit high clay mineral content and well-developed micro-fractures. After invasion by drilling fluid, they are prone to physicochemical interactions, leading to a significant decrease in the strength of the surrounding rock. During drilling operations, occurrences such as wellbore enlargement, cavings, and even collapses have been frequent, making borehole stability an increasingly prominent issue. Currently, there is a lack of systematic understanding regarding the microscopic hydration mechanism and macroscopic mechanical response laws of mudstone in Block S. Therefore, this section systematically analyzes the mineral composition of mudstone and the impact of mudstone hydration on rock mechanical characteristics through X-ray diffraction experiments, triaxial compressive strength tests, and rock immersion triaxial stress experiments, providing an experimental basis for borehole stability control.

### 2.1. Mineral composition analysis of mudstone formation in Block S

**Fig 1:**
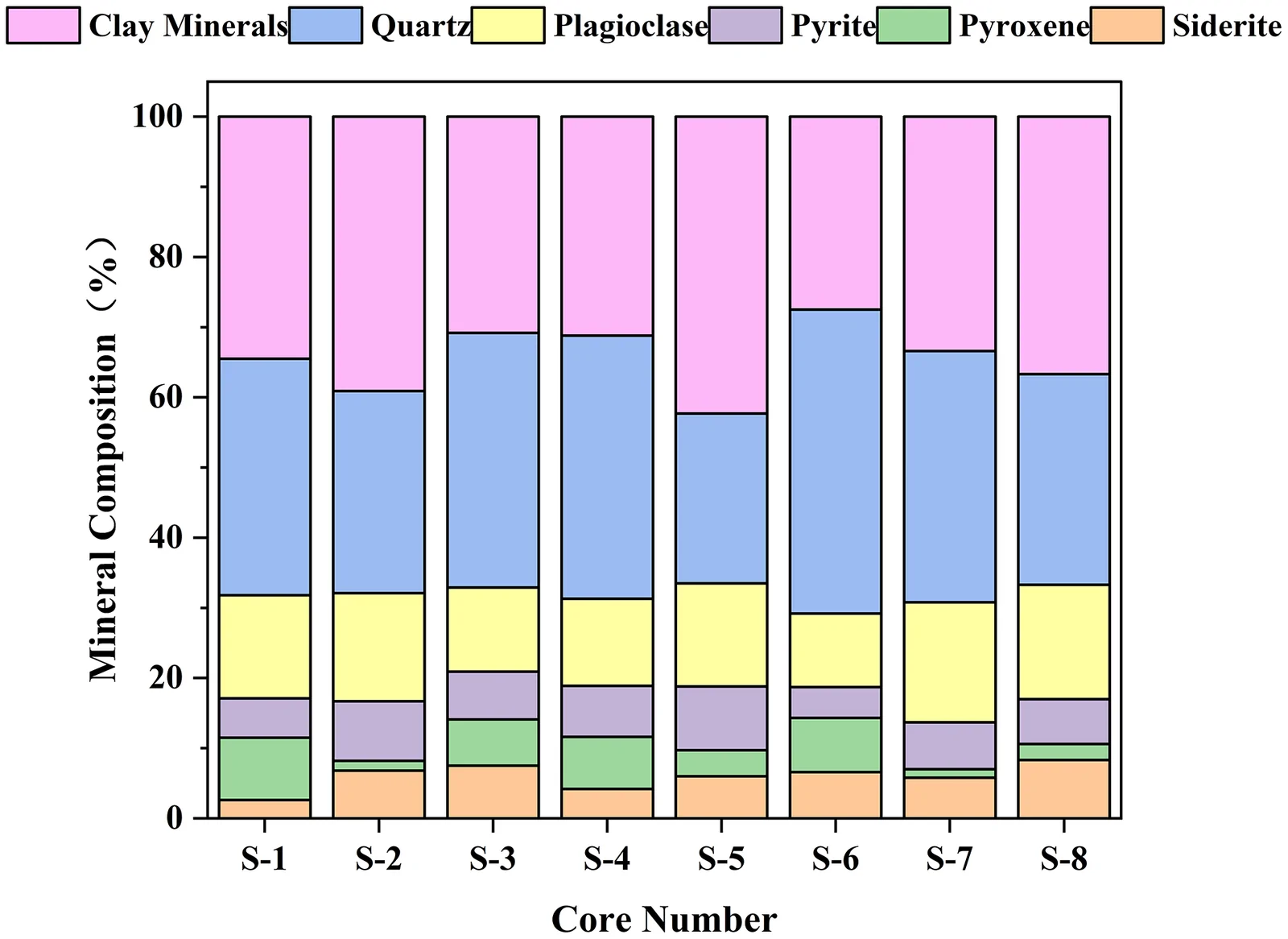
X-ray diffraction analysis results for total mineral composition of mudstone cores.

**Fig 2:**
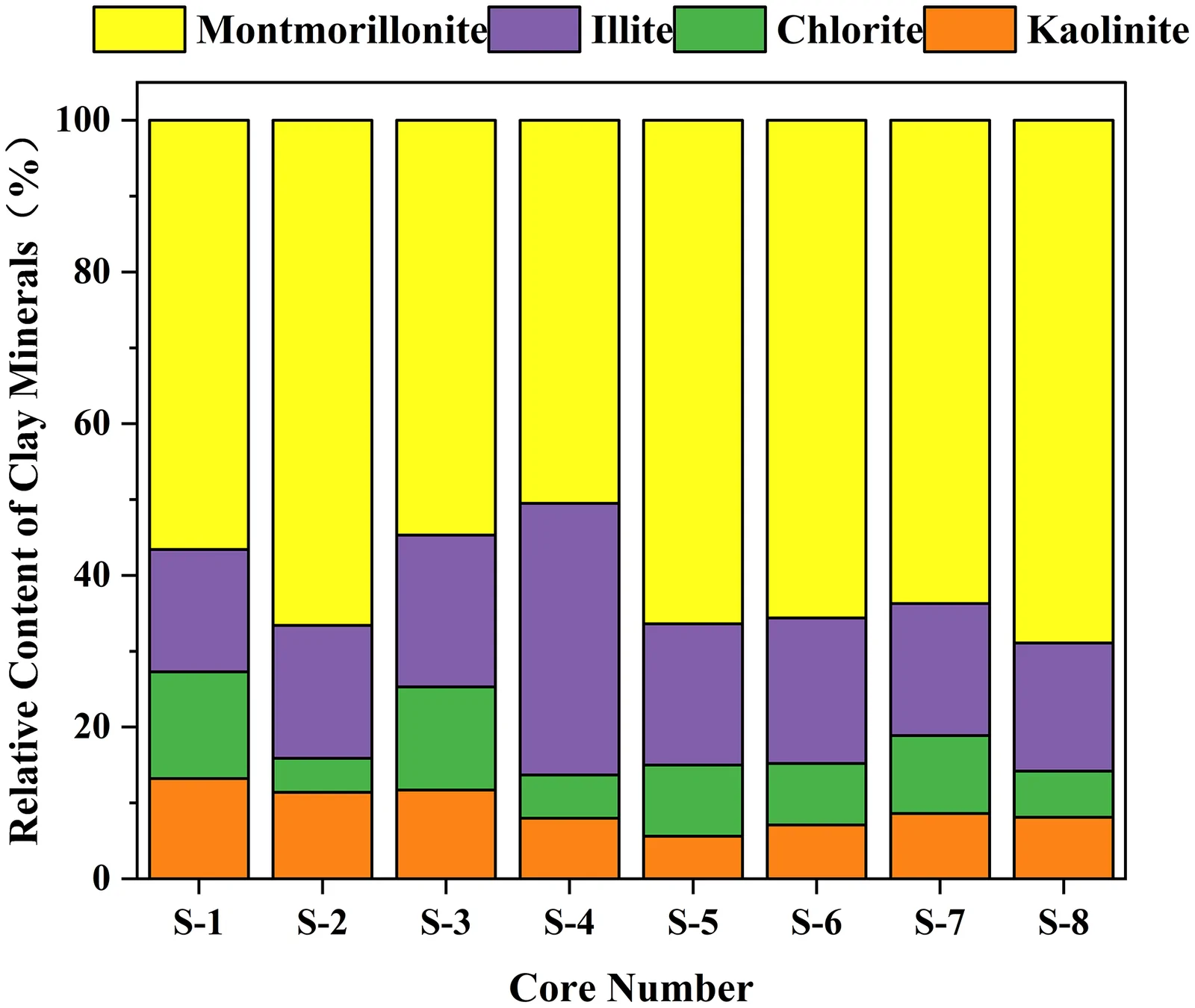
Analysis results of clay mineral relative abundances.

To determine the mineral composition and content of Block S, X-ray diffraction (XRD) experiments were conducted on cuttings from 8 core samples. Based on the diffraction patterns obtained from the experiments, the peaks in each set of patterns were statistically analyzed. The results of the mudstone mineral composition analysis and the analysis of relative clay mineral content are shown in Figs 1 and 2, respectively.

As shown in Fig 1, the average clay mineral content in the mudstone of this area is approximately 34.44%, and quartz is about 33.7%, both being the main components. Studies indicate that high quartz content is positively correlated with the rock brittleness index, which significantly increases the development degree of micro-fractures; whereas clay minerals undergo hydration upon contact with water, leading to rock strength attenuation [20]. Therefore, the synergistic effect of high clay mineral and quartz content is an important internal cause for the instability-prone nature of this mudstone formation.

To further reveal the essence of its strong water sensitivity, a quantitative analysis of the clay mineral components was conducted. The results in Fig 2 show that montmorillonite is overwhelmingly dominant among the clay minerals, with its relative content as high as 50.5% to 68.9%, averaging 61.6%; the contents of illite, kaolinite, and chlorite are relatively low. Montmorillonite possesses a typical 2:1 type expandable crystal lattice structure and an extremely large specific surface area, making it the clay mineral with the strongest hydration expansion capacity. When external fluid invades, montmorillonite layers adsorb a large number of water molecules, generating significant interlayer expansion stress. This is the most core mineralogical mechanism triggering the sharp decrease in rock strength and borehole instability. Non-expansive clay minerals like illite undergo cement softening upon water contact, further weakening the microstructure.

### 2.2. Comparative analysis of mudstone mechanical property deterioration under different immersion conditions

**Fig 3:**
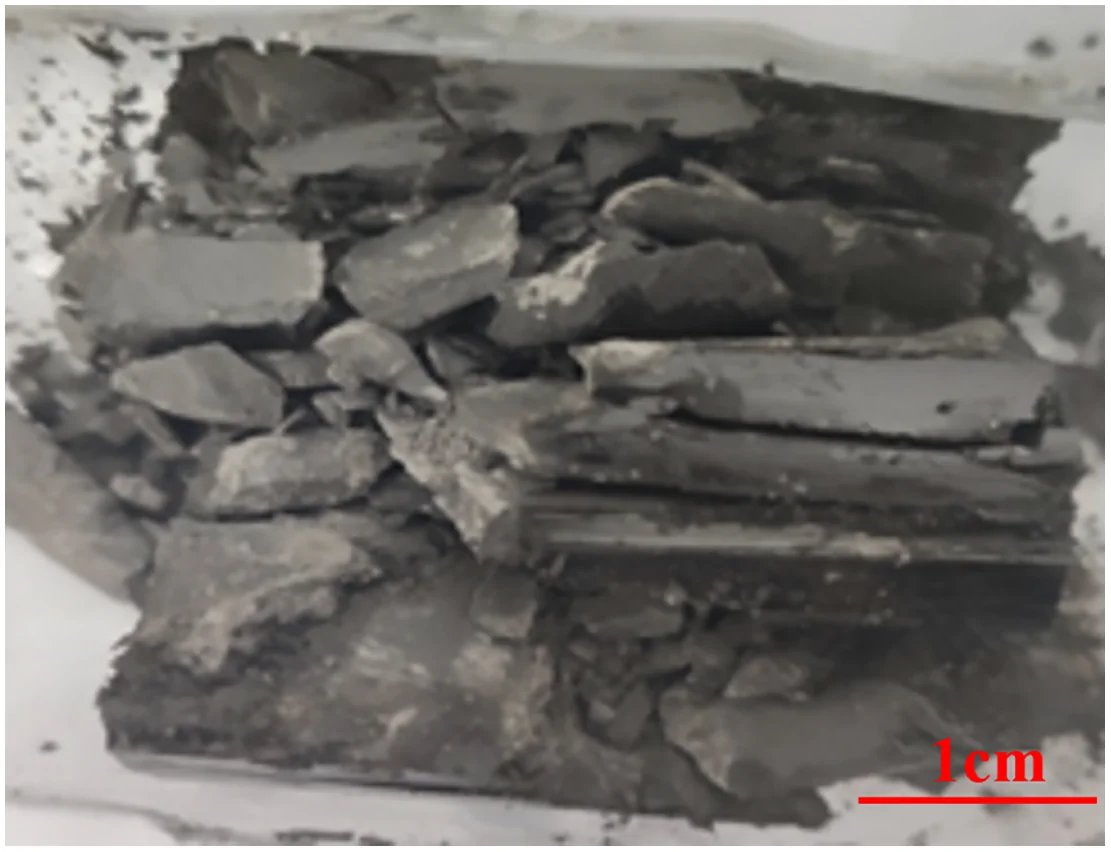
Part of core fracture morphology.

To quantitatively evaluate the deterioration effect of different immersion regimes (fluid type and duration) on mudstone mechanical properties, this section conducts a comparative analysis using original state parameters as a benchmark, based on triaxial mechanical tests on immersed rock samples. Core samples from Block S were prepared into standard cylindrical specimens with a length of 5 cm and a diameter of 2.5 cm. A computer-controlled triaxial stress testing machine was used to perform triaxial compressive strength tests on the core specimens. The fracture morphology of some cores is shown in Fig 3. The parameters of the non-immersed mudstone, such as elastic modulus, Poisson’s ratio, compressive strength, cohesion, and internal friction angle, are listed in Table 1. For each condition, three parallel core samples were tested under confining pressures of 0 MPa, 15 MPa, and 30 MPa, respectively, and the Mohr–Coulomb strength parameters were derived by linear fitting of the peak strength data.

**Table 1:** Test results of conventional triaxial mechanical parameters.

Core number	Elastic modulus (GPa)	Poisson’s ratio	Compressive strength(MPa)	Cohesion(MPa)	Internal friction angle(°)
S-1	30.45	0.24	29.51	25.81	18.64
S-2	26.97	0.25	54.97	26.90	16.28
S-3	22.64	0.21	51.46	28.28	16.55
S-4	18.77	0.26	69.33	26.52	17.73
S-5	34.31	0.24	31.25	22.66	20.12
S-6	29.46	0.23	56.45	20.99	24.45
S-7	24.92	0.27	50.78	20.37	23.76
S-8	15.60	0.24	65.64	17.23	25.69

**Fig 4:**
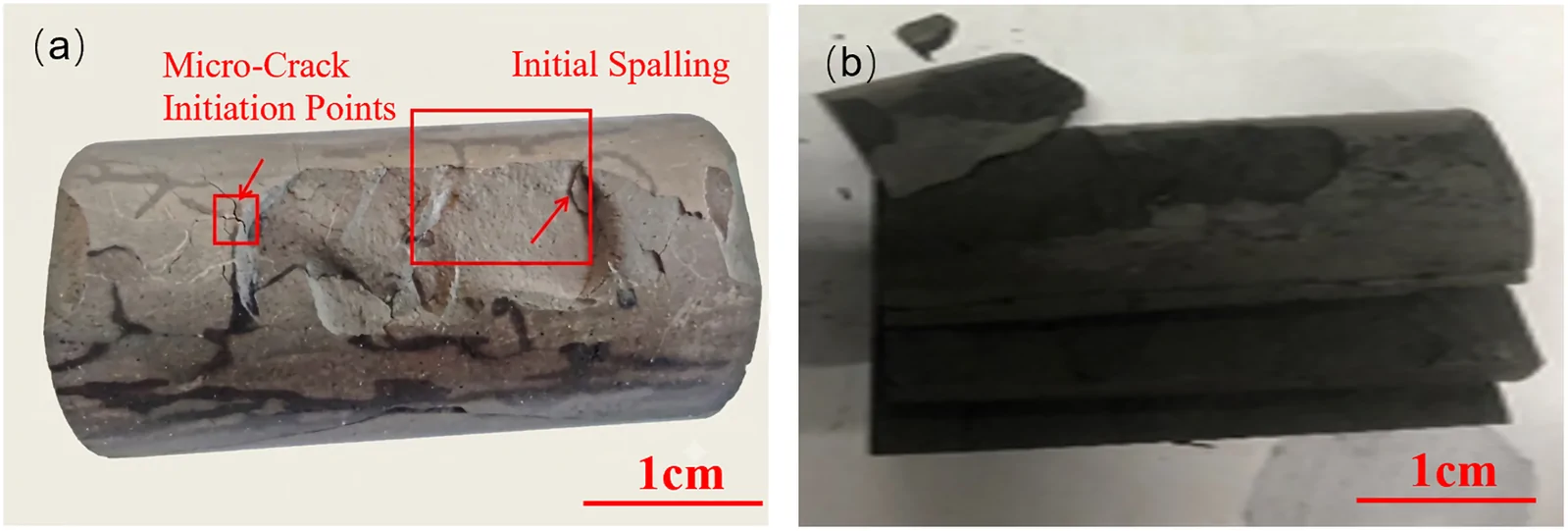
Images of core S-2 after immersion in freshwater for varying durations. **(a)** Core S-2 fell off in 8 hours; **(b)** Core S-2 fractured within 24 hours.

Three standard core specimens from Block S were selected for immersion experiments. Core S-1 and Core S-2 were placed in fresh water, and Core S-5 was placed in a water-based drilling fluid. The water‑based drilling fluid used for immersing core S‑5 was a typical polymer drilling fluid with the following main components: 4% bentonite, 0.3% carboxymethyl cellulose (CMC) as fluid‑loss reducer, 0.2% polyanionic cellulose (PAC) as viscosifier, and 2% KCl as inhibitor. Its measured density was 1.28 g/cm^3^, pH 9.2, and API fluid loss 6.8 mL/30 min, which are consistent with the field‑used fluid in Block S. To prevent core rupture during immersion, Core S-1 and Core S-5 were protected with heat shrink tubing, while Core S-2 was left unprotected. As shown in Fig 4, Core S-2 exhibited significant spalling after 8 hours of immersion in water and fractured after being taken out at 24 hours.

Triaxial compressive strength tests were conducted on Core S-1 and Core S-5 with confining pressures of 0 MPa, 15 MPa, and 30 MPa, respectively. The mechanical parameters of the rock after immersion obtained from the experiments are shown in Table 2.

**Table 2:** Comparison table of mechanical parameters of rock after soaking.

Core number	Confining pressure (MPa)	Soaking type	Poisson’s ratio	Compressive strength (MPa)	Cohesion (MPa)	Internal friction angle(°)
S-1	0	Freshwater	0.30	13.24	12.62	6.38
	15		0.31	25.67		
	30		0.29	32.19		
S-5	0	Water-based drilling fluid	0.28	20.31	15.07	14.98
	15		0.27	29.75		
	30		0.27	39.42		

Both experimental phenomena and changes in mechanical parameters indicate that immersion in fresh water and water-based drilling fluid significantly reduces the mechanical property parameters of mudstone. Compared to the original state of the mudstone, the Poisson’s ratio of mudstone after fresh water immersion increased by 20%, compressive strength decreased by 55.13%, and cohesion decreased by 51.10%. For mudstone immersed in water-based drilling fluid, the Poisson’s ratio increased by 12.5%, compressive strength decreased by 35.01%, and cohesion decreased by 33.50%.

The core exhibited significant progressive failure during the immersion process. As shown in Fig 4, the unprotected Core S-2 showed obvious blocky spalling after only 8 hours of immersion in fresh water and completely fractured after 24 hours. This indicates that water molecule invasion does not uniformly weaken the rock but preferentially acts along micro-fractures and mineral interfaces, causing the rock structure to delaminate from the surface inward. Combined with the mineral composition analysis in Section 2.1, a possible reason for this phenomenon lies in the high montmorillonite content in the mudstone. Montmorillonite, as a strongly water-sensitive clay mineral, undergoes continuous, staged expansion of its interlayer structure after water molecule invasion. This microscopic lattice expansion stress translates macroscopically into local tensile stress, continuously exacerbating the propagation and interconnection of micro-fractures. Simultaneously, the invading free water generates pore pressure effects within the fractures, further reducing effective stress and the cementation strength between particles. The coupling effect of montmorillonite hydration expansion and fracture water pressure leads to the continuous deterioration of core strength with increasing immersion time, ultimately resulting in instability and failure. This also explains why, even with water-based drilling fluid immersion, although the degree of mechanical parameter deterioration is less severe than with fresh water, the overall trend remains consistent with the mineral hydration mechanism.

## 3. Molecular dynamics model of mudstone under hydration

### 3.1. Construction and optimization of the molecular dynamics model for mudstone montmorillonite

Although the mudstone contains minor amounts of illite, kaolinite, and chlorite, montmorillonite is the dominant clay mineral and possesses by far the strongest hydration expansion capacity. Therefore, montmorillonite is selected as the representative mineral for the molecular dynamics simulation to capture the primary microscopic mechanism governing mudstone hydration and mechanical deterioration. The water absorption and expansion rate of sodium-based montmorillonite is far higher than that of calcium-based montmorillonite, and its rock mechanical parameters decrease rapidly [21]. Therefore, the model in this paper selects sodium-based montmorillonite, which exhibits faster reaction rates and more pronounced swelling, as the research object. The XRD experimental results in Section 2.1 indicate that Block S is rich in iron elements, and the probability of Fe^2+^ substitution in the lattice is greater than that of Mg^2+^ substitution. Therefore, the montmorillonite molecular formula established in this paper, conforming to Block S, is: $\left({\text{Fe}}_{\text{y}}^{\text{2+}}{\text{-nH}}_{\text{2}}\text{O}\right)\left({\text{Al}}_{\text{2-y}}^{\text{3+}}{\text{Fe}}_{\text{y}}^{\text{2+}}\right){\text{Si}}_{\text{4}}^{\text{4+}}{\text{O}}_{\text{10}}{\left(\text{OH}\right)}_{\text{2}}$. In the formula, y represents the charge number. When y takes the minimum value of 0.19, the ratio of iron to aluminum content is 1:9; when y takes the maximum value of 0.6, the ratio of iron to aluminum content is 1:2.36. The ratio of aluminum to iron content within this interval can ensure the stability of the montmorillonite molecular structure. This paper adopts a ratio of 1:7 for the substituting iron to aluminum elements. The 1:7 ratio yields an interlayer charge number of approximately 0.38, which falls within the typical range of 0.2–0.6 for montmorillonite and ensures both structural stability and computational efficiency, while accurately reflecting the iron-rich characteristics of the Block S formation. Table 3 shows the spatial coordinates of different types of atoms in the established montmorillonite molecular unit cell.

**Table 3:** Spatial coordinates of different types of atoms in montmorillonite molecular cell.

Element	X(Å)	Y(Å)	Single layer	Double layers	Three layers
			Z1(Å)	Z2(Å)	Z3(Å)
Si	0.472	1.510	9.580	12.580	15.580
Na^+^	0	4.530	6.250	9.250	12.250
Al	0	3.020	12.500	15.500	18.500
O	0.122	0.000	9.040	12.040	15.040
O	−0.686	2.615	9.240	12.040	15.240
O	0.772	1.510	11.200	14.200	17.200
O(OH)	0.808	4.530	11.250	14.250	17.250
H(OH)	−0.103	4.530	10.812	13.812	16.812

**Fig 5:**
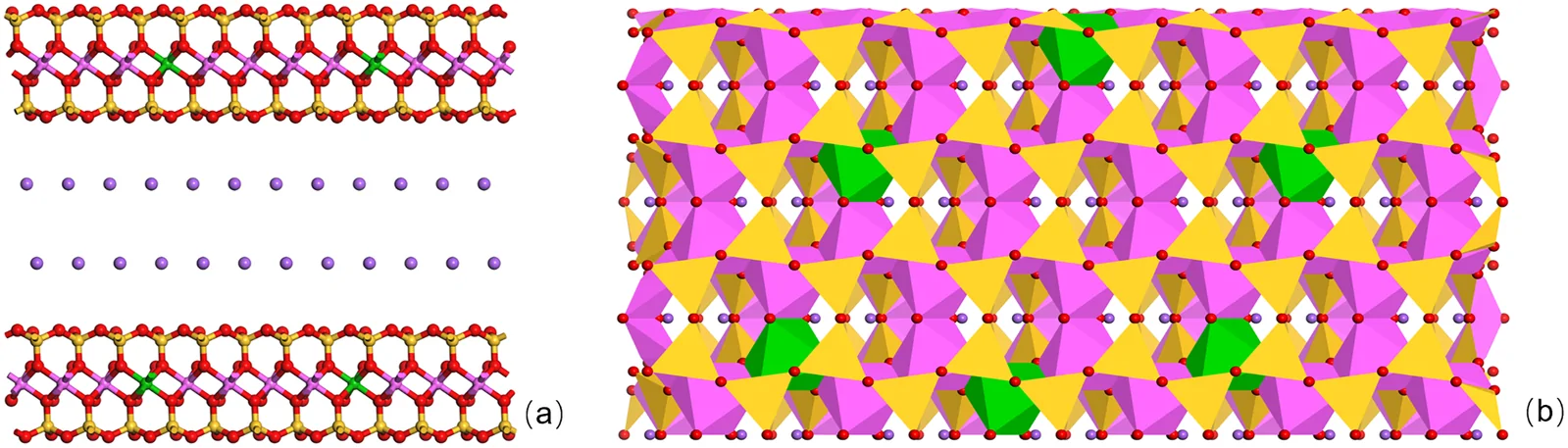
Iron element substituted montmorillonite molecule diagram. **(a)** Iron substituted montmorillonite molecule**; (b)** octahedral substitution model.

A schematic diagram of the supercell model for the Fe^2+^ -substituted montmorillonite molecule is shown in Fig 5(a), and its octahedral substitution model schematic is shown in Fig 5(b).

**Fig 6:**
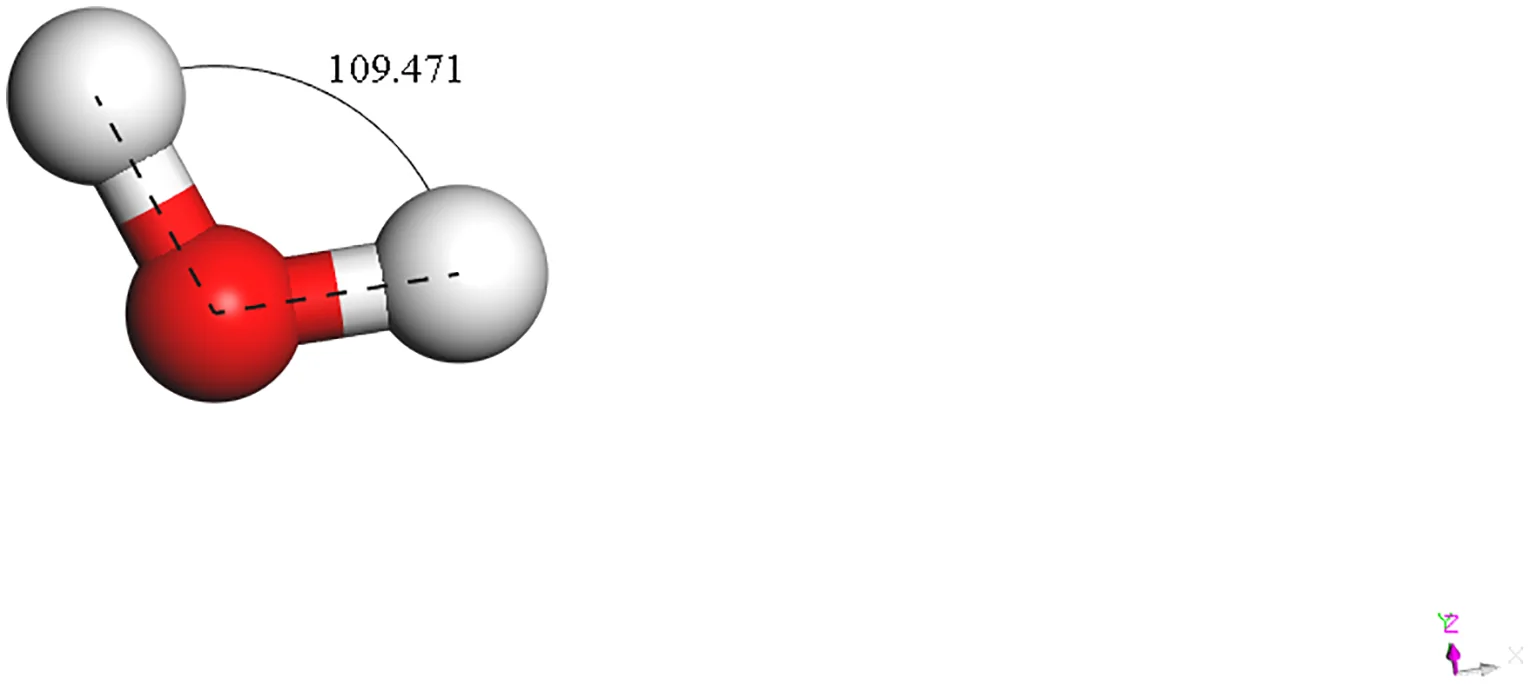
Schematic diagram of water molecular model.

The SPC/E model [22] was selected for water molecules in this paper, with a bond length *r*_*0*_ = 1 Å and a bond angle *θ* = 109.47°. The simulation diagram of a water molecule is shown in Fig 6.

Due to the large number of atoms within the model unit cell and the high system energy, atomic energy optimization is necessary to prevent calculation errors and obtain the optimal solution for the model energy: (1) The average force threshold for atoms is 0.005 kcal/mol/Å; (2) The energy deviation should not exceed 1 × 10^−4^ kcal/mol, which helps to determine whether the energy has been fully optimized; (3) The convergence criterion for atomic displacement is primarily evaluated by mean square displacement, which should be less than 5 × 10^−5^ Å to ensure atoms are adjusted to their optimal positions; (4) The root mean square stress of the crystal structure is limited to within 5 Pa; (5) To reasonably control the computational scope and efficiency, a cutoff radius of 8 Å is set; (6) Regarding the charge assignment strategy, the charge equilibration method is employed to achieve charge balance within the system; (7) The Ewald method is used to calculate long-range Coulomb electrostatic forces; (8) The van der Waals forces are solved using the Atom-Based method.

### 3.2. Model validation

To verify the reliability and accuracy of the model and to evaluate whether the optimized model can achieve the expected results, this paper compares with experiments conducted by previous researchers. The controlled variable method is used to investigate the change in interlayer spacing during hydration. The temperature, pressure, and water content settings of the model are kept consistent with previous studies, as detailed in Table 4.

**Table 4:** Parameter settings for model validation.

Number of water molecules	Pressure	Temperature	Reference group	
			Boek	Jiafang Xu
32	0.1MPa	298K	32	32
64			64	64
96			96	96

**Fig 7:**
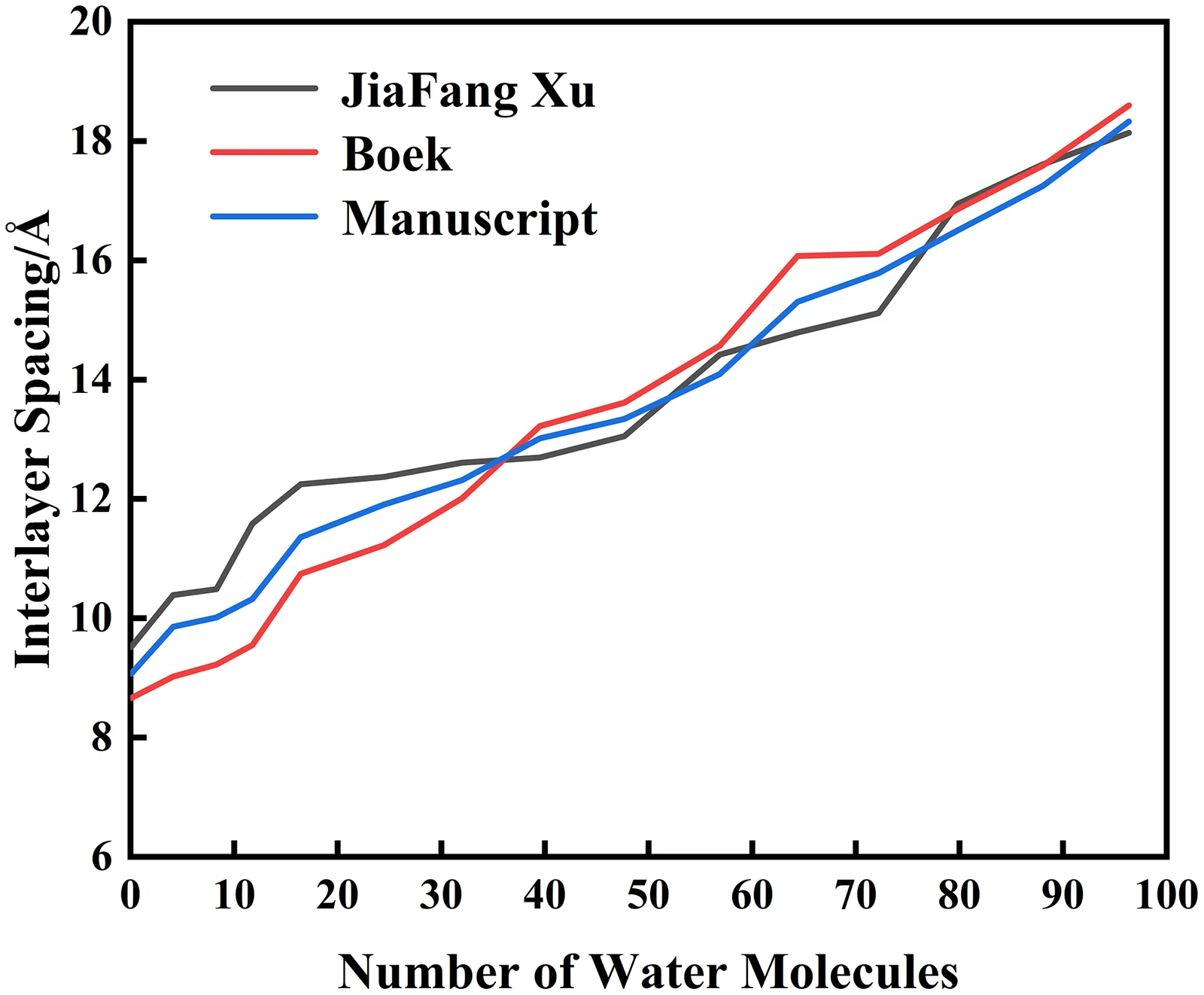
Curve of interlayer spacing changing with the number of water molecules.

The variation curve of montmorillonite molecular interlayer spacing with increasing number of water molecules is shown in Fig 7.

**Fig 8:**
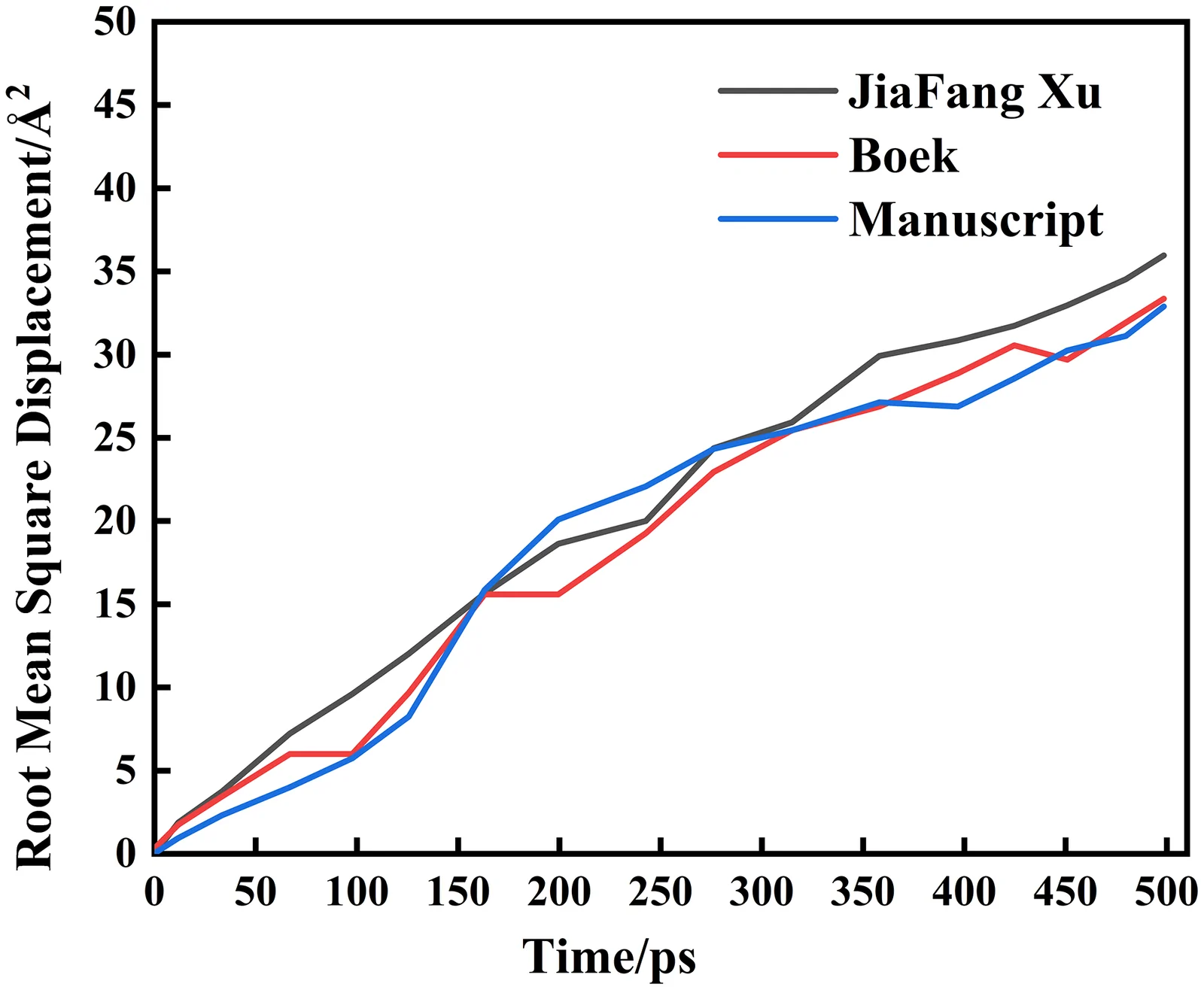
Comparison of root mean square displacement.

**Fig 9:**
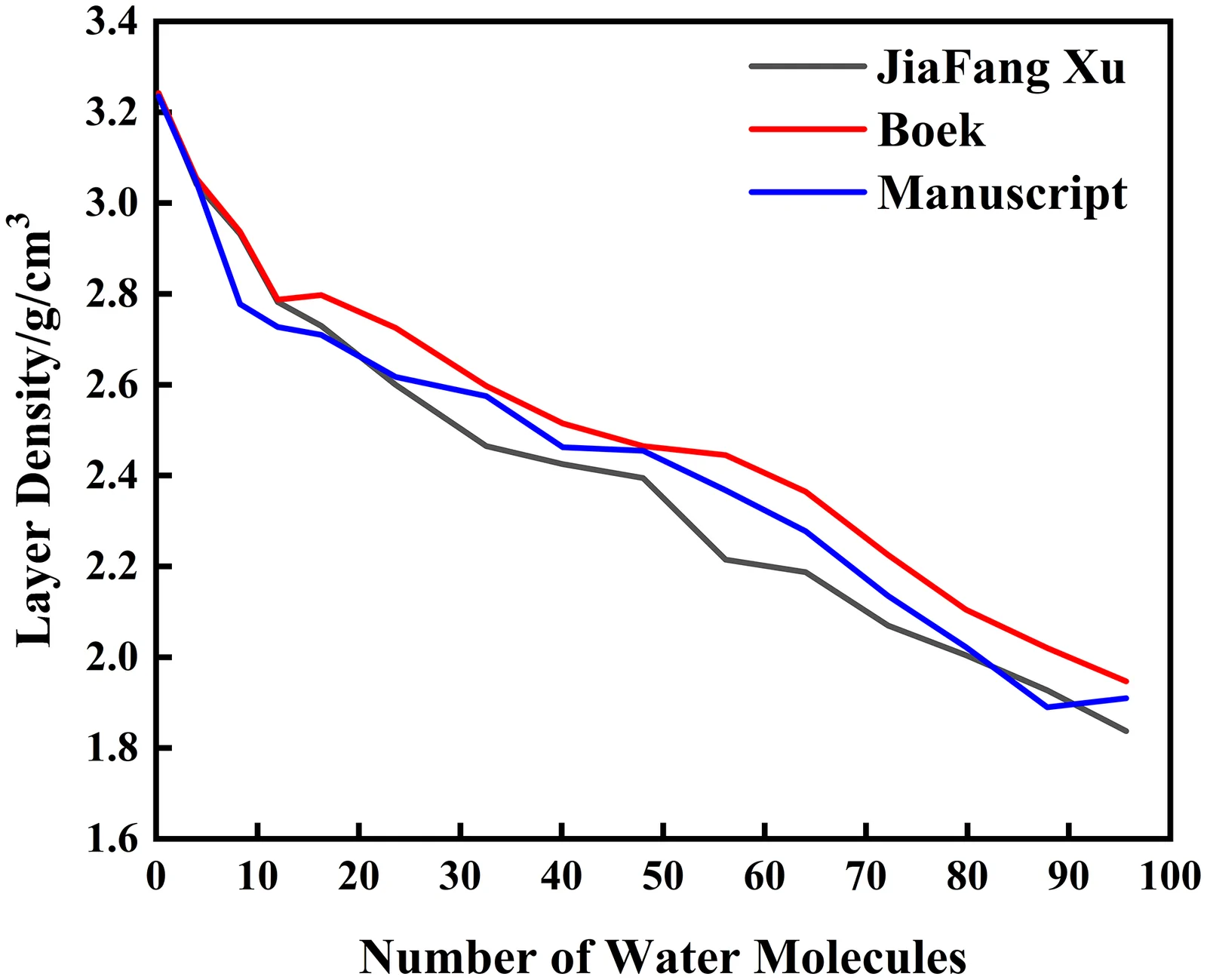
Variation in layer density with the number of water molecules.

As shown in Fig 7, the interlayer spacing of montmorillonite shows a significant increasing trend with rising water content. The deviations between the experimental results of this paper and those of Xu Jiafang [23] and Boek [24] are 6.89% and 7.94%, respectively, falling within a reasonable error range, validating the rationality of the simulation design. To further verify the model’s correctness, it is necessary to continue comparing the mean square displacement and layer system density of the three models. The comparison results are shown in Figs 8 and 9.

Based on the comparative analysis of Figs 8 and 9, the errors for the sodium ion root mean square displacement results compared to the experiments of Xu Jiafang and Boek are 6.9% and 6.67%, respectively. The errors for the layer density results compared to their experiments are 7.07% and 3.7%, respectively. The simulation results are approximately the same as those of previous studies. Therefore, it can be concluded that the sodium-based montmorillonite model established in this paper possesses high accuracy and reliability.

## 4. Molecular dynamics simulation of montmorillonite hydration process

### 4.1. Evolution of interlayer spacing and its influence on water film structure formation

**Fig 10:**
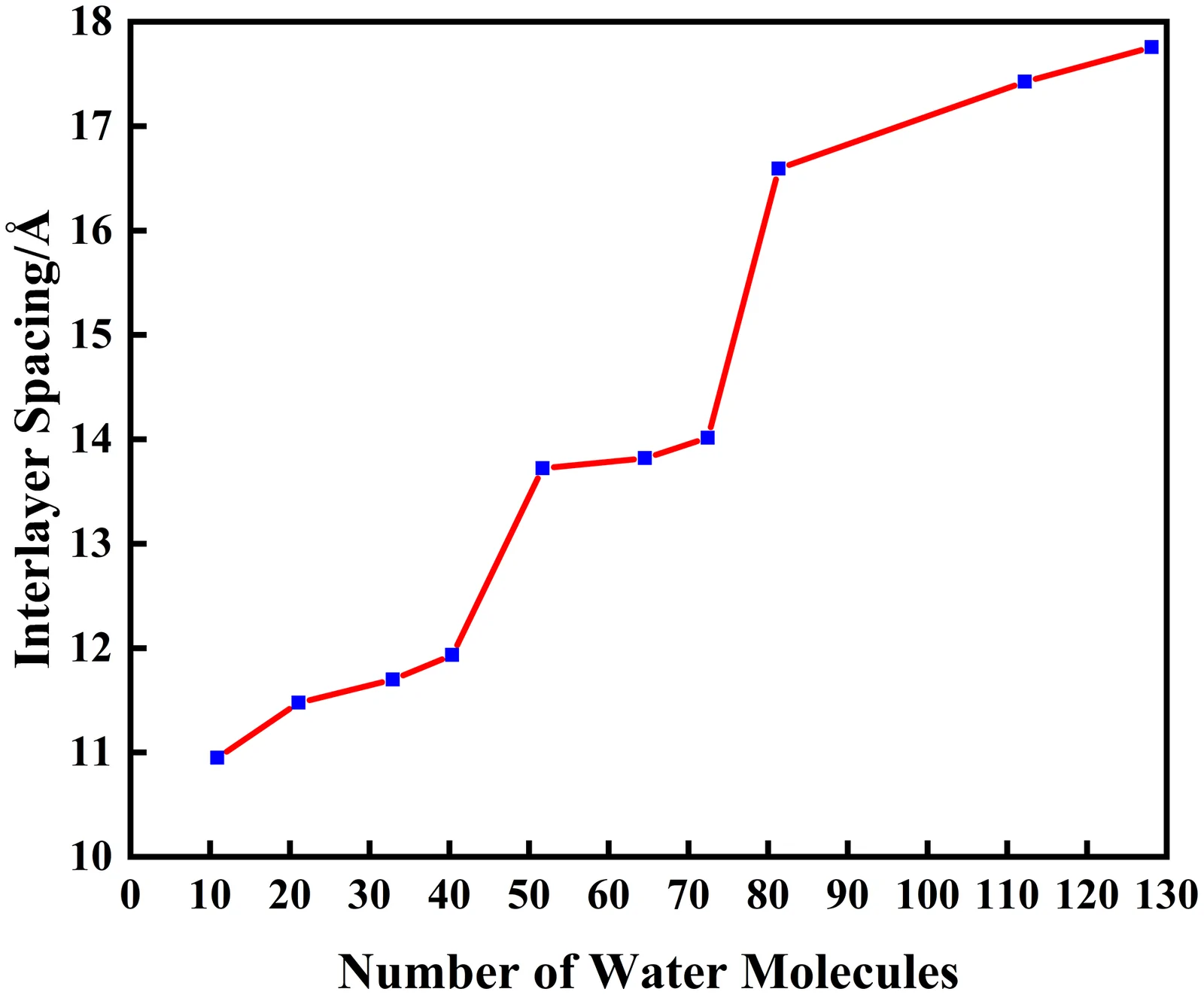
The interlayer spacing of iron-substituted montmorillonite varies with the number of water molecules.

The initial simulation conditions were set as: temperature 25°C, pressure 0.1 MPa. By gradually increasing the number of water molecules, the microscopic mechanism of interlayer spacing changes with increasing water content was analyzed. Using the above model, the curve of Fe-substituted montmorillonite molecular interlayer spacing varying with water molecule content can be obtained, as shown in Fig 10.

**Fig 11:**
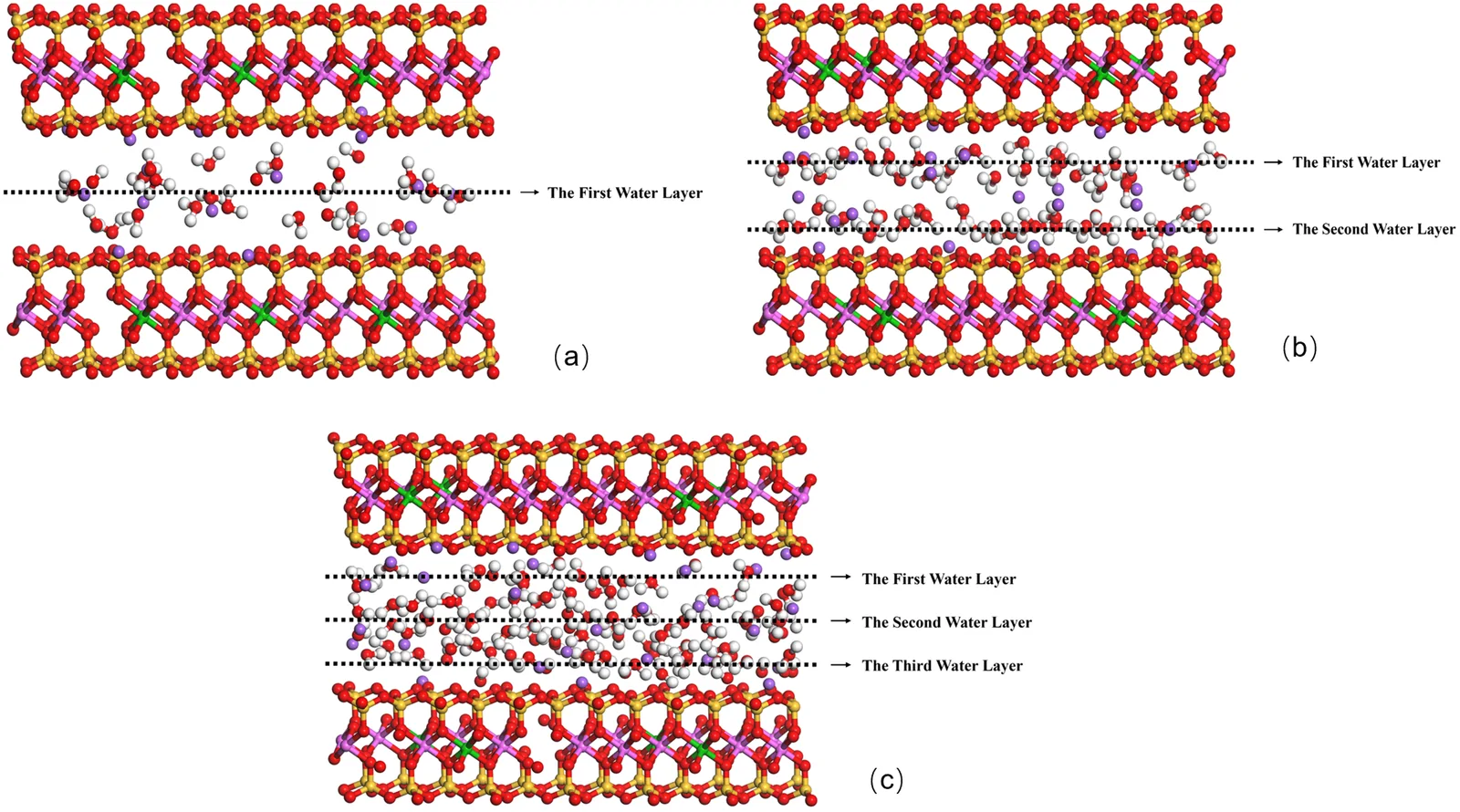
Simulation results under different numbers of water molecules. **(a)** Single layer water molecules; **(b)** Double layer water molecules; **(c)** Three layer water molecules.

As shown in Fig 10, the interlayer spacing of the Fe-substituted montmorillonite molecule gradually increases with the addition of water molecules, and its water absorption expansion is also significantly enhanced. To further quantify the continuous change characteristics of interlayer spacing during the actual hydration process, this study further simulated the relationship between the relative proportion of water molecules and the interlayer spacing. The results are shown in Fig 11.

**Fig 12:**
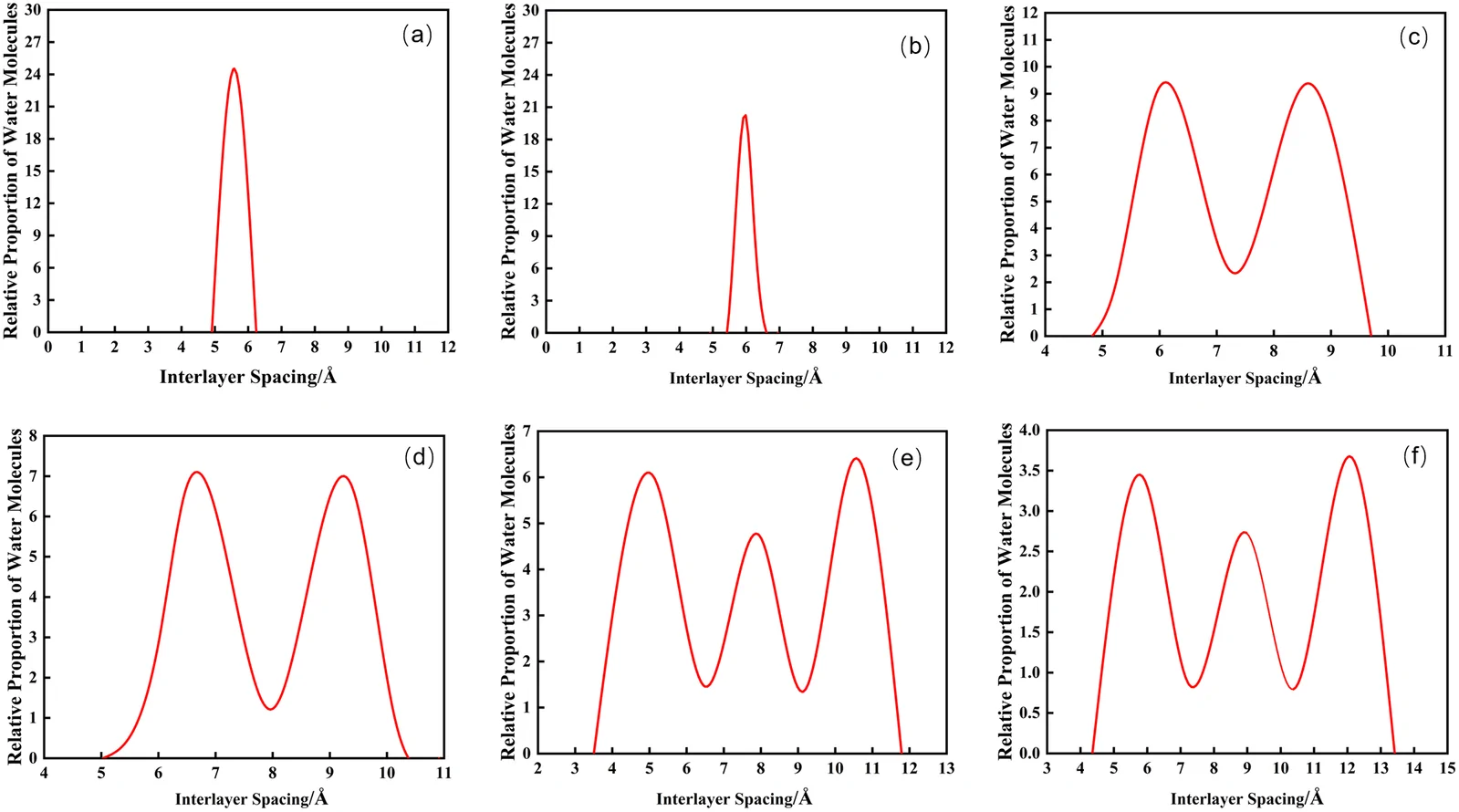
Interlayer spacing and water molecular concentration diagram. (a) 12 water molecular interlayer spacing; (b) 32 water molecular interlayer spacing; (c) 54 water molecular interlayer spacing; (d) 70 water molecular interlayer spacing; (e) 102 eater molecular interlayer spacing, (f) 124 water molecular interlayer spacing.

As shown in Fig 11, the montmorillonite interlayer hydration process exhibits significant staged expansion characteristics. Constrained by the electrostatic field within the interlayer, water molecules preferentially adsorb at the midline position of the interlayer region, forming a single-layer ordered water film, with the interlayer spacing relatively increasing, as shown in Fig 11 (a). As the number of water molecules invading the montmorillonite molecular interlayer region continues to increase, influenced by the combined electrostatic attraction from the upper and lower silicon-oxygen layers, water molecules spontaneously stratify, forming a double-layer water film structure. At this point, the interlayer spacing increases significantly, with an increment of 3 Å to 5 Å, as shown in Fig 11 (b). When the number of water molecules further increases, the interlayer water molecules are stably distributed in the form of a triple-layer thick water film, and the interlayer spacing increases to a maximum value of 10 Å to 15 Å. At this stage, the montmorillonite molecule reaches its maximum expansion deformation along the Z-axis direction, as shown in Fig 11 (c). To systematically study the quantitative relationship between the number of water molecules and the law of water film formation, the simulation was used to separately vary the number of water molecules invading the interlayer region. The results regarding the positional regularity of water film formation are shown in Fig 12.

Combining the corresponding relationship between the number of water molecules and the interlayer spacing in Figs 11 and 12, the following conclusions can be drawn: (1) When the number of water molecules participating in the reaction is 10–32, the curve exhibits a single peak, indicating that only a single-layer water film forms inside the montmorillonite molecule. The interlayer spacing of the montmorillonite molecule at this stage is 6.24 Å to 6.96 Å. (2) When the number of water molecules participating in the reaction is 54–70, two broad peaks appear in the graph, indicating the formation of two water film layers inside the montmorillonite molecule, and the water films are relatively thick. The montmorillonite molecular interlayer spacing gradually increases, reaching 9.70 Å to 10.91 Å. (3) When the number of water molecules participating in the reaction is 102–124, three broad peaks appear, indicating the formation of three water film layers inside the montmorillonite molecule. Due to the attractive effect of the montmorillonite molecule on water molecules, the thickness of the upper and lower water films is greater, while the middle water film is relatively thinner. The montmorillonite molecular interlayer spacing at this stage is 11.79 Å to 13.42 Å.

The simulation results indicate: As the number of water molecules participating in the reaction increases, the interlayer spacing of the Fe-substituted montmorillonite molecule significantly enlarges, and structural expansion is evident, leading to a nonlinear increase in interlayer spacing, which affects its original stable structure.

### 4.2. Characteristics of particle distribution within the interlayer region

**Fig 13:**
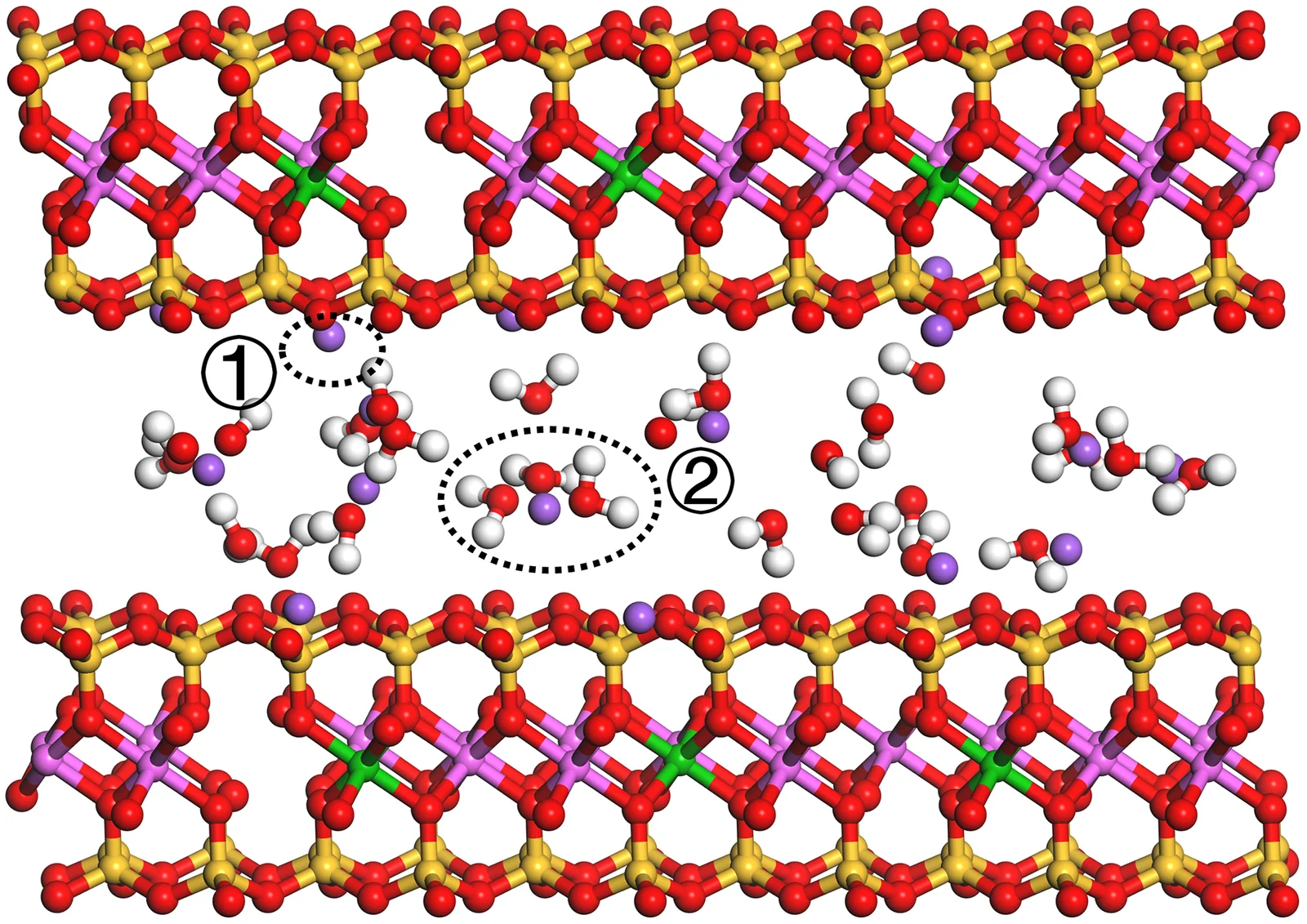
Distribution of Na^+^ in monolayer water molecules.

During the hydration reaction of montmorillonite molecules, hydrogen bonds mainly originate from water molecules, and cations mainly originate from Na⁺ within the montmorillonite molecule. As shown in Fig 13, taking the Na⁺ distribution pattern under a single-layer water molecule as an example, after the hydration reaction, the existence forms of Na⁺ within the interlayer region are mainly divided into two types: The first is distributed on the surface of the silicon-oxygen hexagonal rings of the montmorillonite molecule, forming a typical inner-sphere coordination structure with the surface oxygen atoms; The second is distributed within the interlayer region, achieving balance by attracting coordinating water molecules. Na^+^ attracts three surrounding water molecules, all of which exhibit the characteristic of hydrogen atoms facing outward and oxygen atoms oriented towards Na^+^, together with Na^+^ forming hydrated ions. Because the diameter of the formed hydrated ion is much larger than the silicon-oxygen hexagonal rings on the montmorillonite molecule surface, it can only exist within the interlayer region. Simultaneously, a large number of hydrogen bonds in various forms formed within the interlayer region can stably adsorb free water entering the interlayer as bound water. Their interaction force is far greater than van der Waals forces. The formed hydrated ions can stably adsorb on the internal surface of the interlayer region, which is also the key mechanism enabling the formation of stable water molecule layers within the montmorillonite molecular interlayer region.

**Fig 14:**
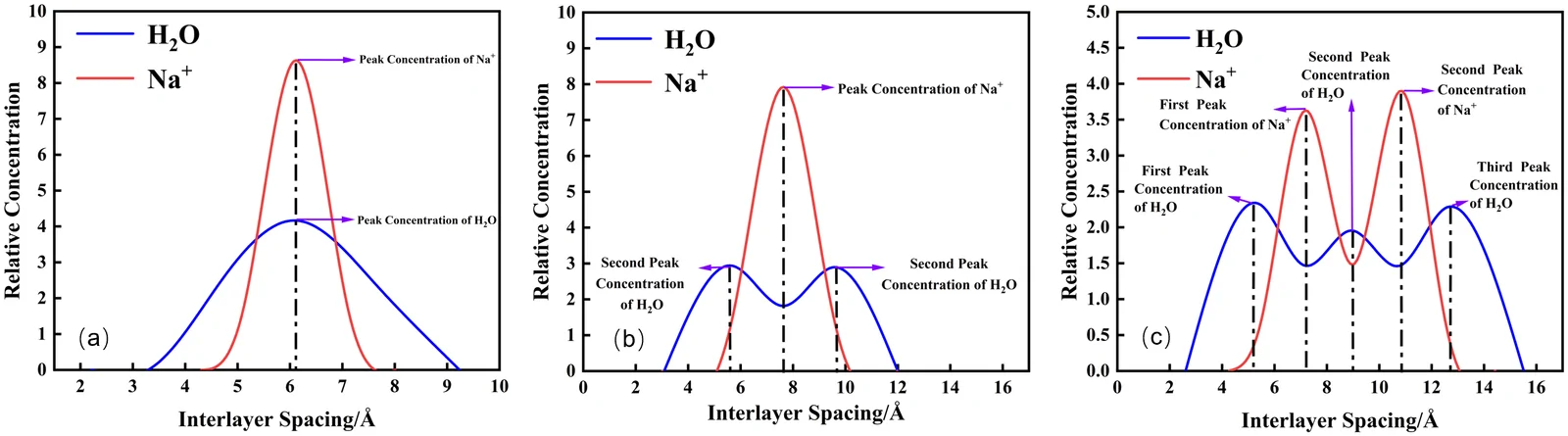
Relationship between H_2_O and Na^+^ concentrations. **(a)** Single layer water molecules; **(b)** Double layer water molecules, **(c)** Three layer water molecules.

Continuing the analysis of the existence forms of Na⁺ in the montmorillonite molecule, it is found that its distribution pattern is related to the water molecule content within the interlayer region. Using the radial distribution function to statistically analyze particle concentration data perpendicular to the Fe-substituted montmorillonite molecular surface [25], relative concentration curves of cations along the normal direction of the (0 0 1) plane were plotted. The motion trajectories of cations and the changes occurring during the hydration process were comprehensively analyzed. Concurrently, water molecules combined around the cations were studied to reveal the stability mechanism of intermolecular attraction. The simulation results are shown in Fig 14.

**Fig 15:**
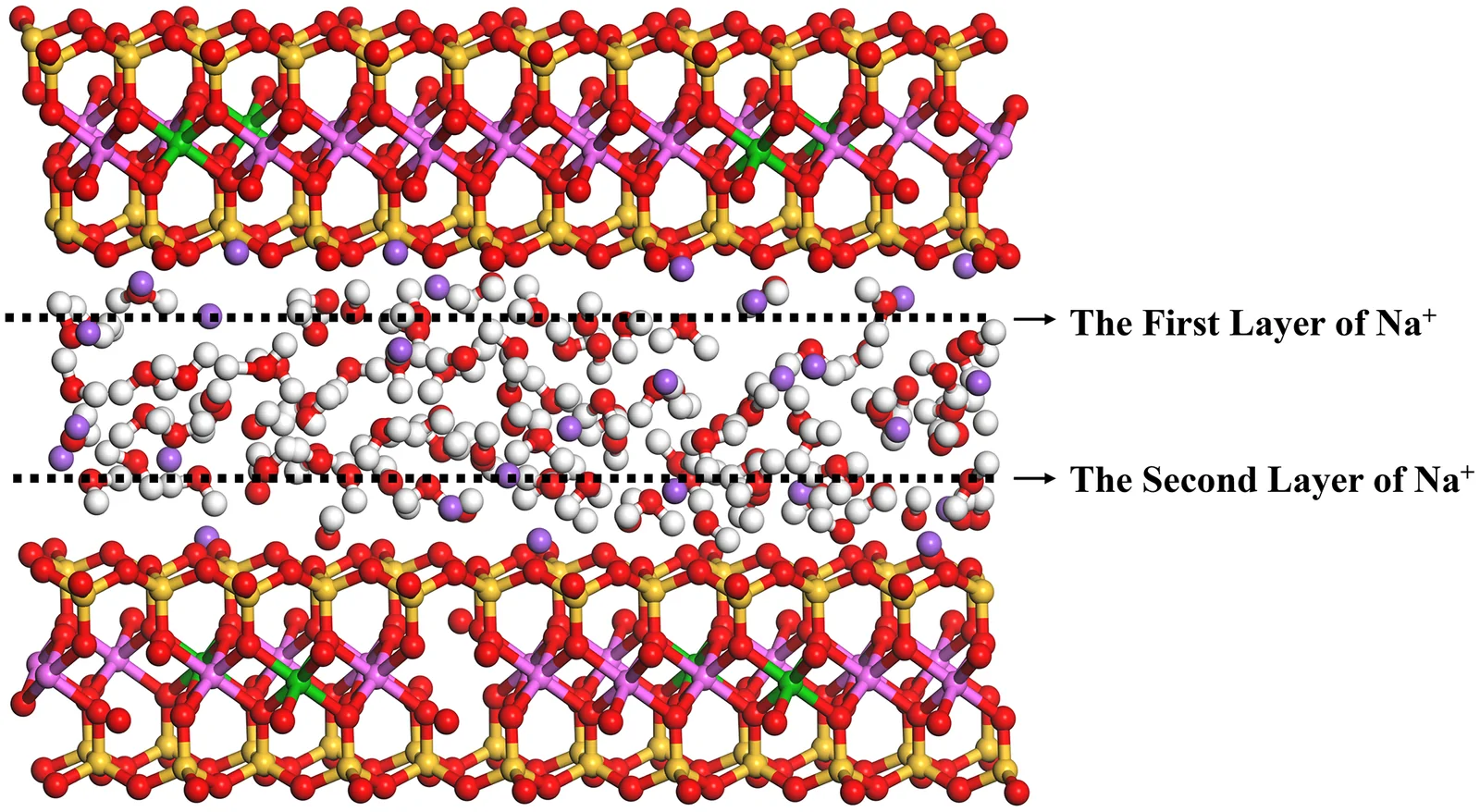
Relative concentration relationship of Na^+^.

According to Fig 14, there are obvious differences between the Na^+^ concentration curve and the water molecule concentration curve distribution. In the initial stage of the hydration reaction, when only a single layer of water molecules exists within the montmorillonite molecule, both the water molecule and Na^+^ curves along the normal direction of the (0 0 1) plane show a single peak. When the interlayer spacing is 6.15 Å, both concentration curves simultaneously reach their maximum values, indicating that the distribution of Na^+^ influences the distribution of internal water molecules, and this distribution is symmetric about the interlayer midline position. As the number of water molecules participating in the hydration reaction increases, the relative concentration curve of water molecules shows two peaks, forming two relatively concentrated and symmetric water molecule layers about the interlayer midline. At this time, Na^+^ moves towards the midline of the interlayer region, and its concentration peak is precisely located at the trough of the water molecule concentration. The interlayer spacing at this stage is 7.9 Å. When the number of water molecules participating in the reaction continues to increase, the concentration curve shows two high-value peaks and one low-value peak, indicating the formation of a “thick-thin-thick” water molecule layer structure within the interlayer region. At this stage, the peak of the Na^+^ concentration distribution curve coincides with the interlayer spacing position at the trough of the water molecule layers, indicating that the Na^+^ layer and the water molecule layers construct an alternating layered structure. The reason for the formation of this structure is the obvious adsorption effect of the negative charge on the montmorillonite molecule surface on Na^+^. As the interlayer spacing increases, when Na^+^ moves towards either side of the crystal lattice edge, the reverse Coulomb force acting on Na^+^ moving away from the center significantly attenuates, causing it to aggregate near the closer lattice side, forming two asymmetric high-concentration Na^+^ layers. At this point, Na^+^ has almost completely detached from the crystal lattice layer and is distributed within the gaps of the three water molecule layers. Figure 15 clearly shows the distribution characteristics of Na^+^ in the three-layer water molecule structure.

The above analysis provides an intuitive understanding of particle position distribution characteristics after hydration through particle trajectories and final configurations, but does not quantitatively characterize particle distribution structures and their interactions. Therefore, the radial distribution function g(r) was used to quantitatively analyze particle configurations and stability under the following four conditions: (1) The radial distribution function between Na⁺ and oxygen atoms in water molecules (Na-O_w_); (2) The radial distribution function between Na⁺ and oxygen atoms on the montmorillonite molecular surface (Na-O_MMT_); (3) The radial distribution function of oxygen atoms in water molecules (O_w_-O_w_); (4) The radial distribution function between oxygen and hydrogen atoms in water molecules (O_w_-H_w_).

**Fig 16:**
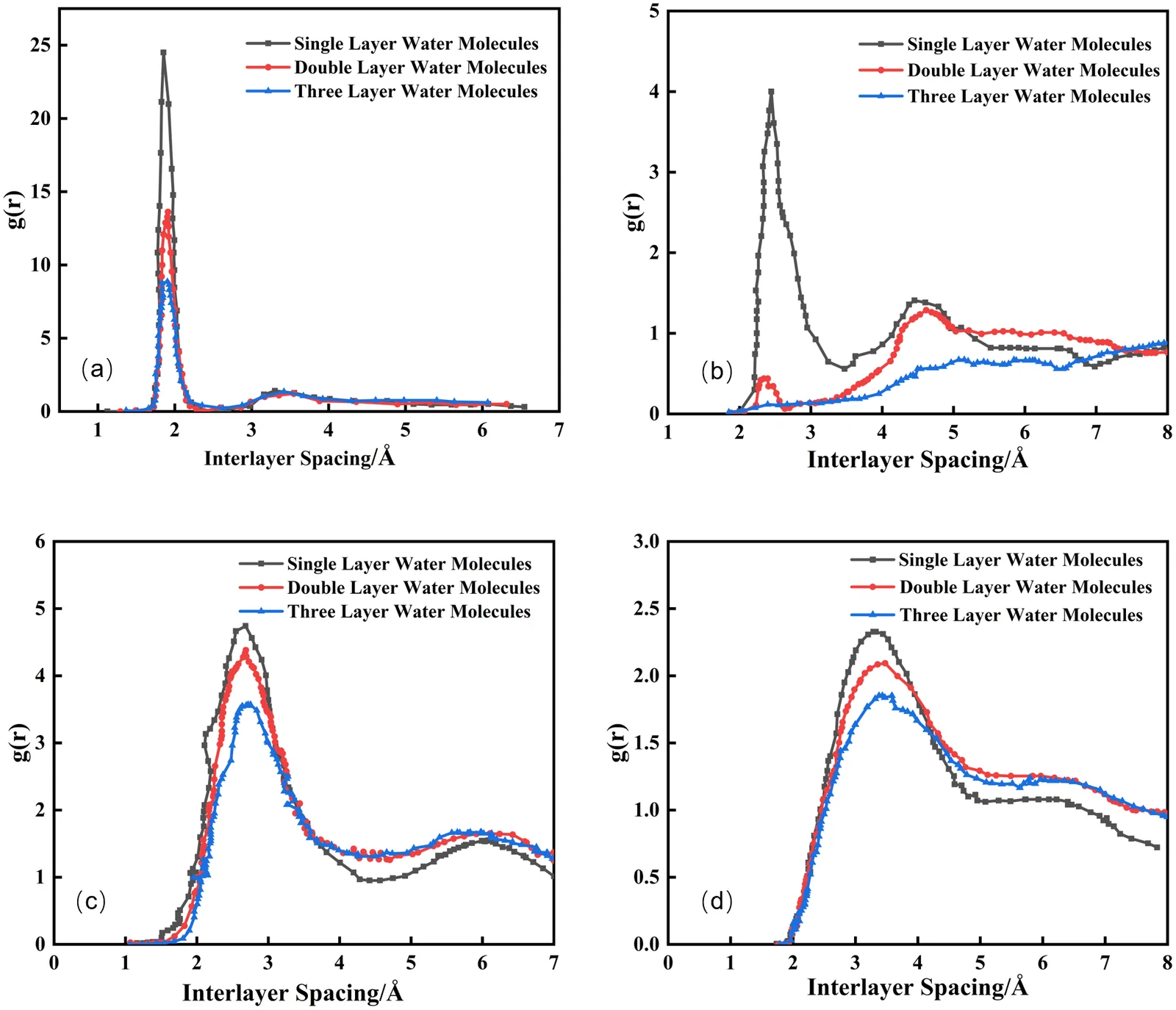
Radial distribution function diagram. (a) Na-O_w_; (b) Na-O_MMT_; (c) O_w_-O_w_, (d) O_w_-H_w_.

As seen in Fig 16 (a), regardless of the number of water molecule layers formed within the interlayer region, the trends of their radial distribution function diagrams are basically the same, all showing a double peak, indicating that Na ⁺ is always combined with two hydration shells of water molecules. However, as the number of water molecule layers increases, the peak value of the radial distribution function gradually decreases, but the curve shape does not change, indicating that the molecular arrangement is not tight, the hydration shells combined around the Na⁺ layer become looser, and the overall internal strength and stability decrease. At this stage, the coordination structure of Na⁺ does not show significant changes. The radial distribution function between Na⁺ and oxygen atoms on the montmorillonite molecule surface, shown in Fig 16 (b), is approximately the same in shape as the function in Fig 16 (a). However, there is a clear difference in the images for the double-layer and triple-layer water molecule cases, where almost no obvious peaks appear. This indicates that most Na⁺ have moved away from the montmorillonite molecule surface, with only a small number of paired chemical bonds remaining, and the ordering of water molecules has already changed. This conclusion is consistent with the finding in Fig 14 that Na⁺ concentration increases with higher water content and gradually moves towards the midline of the interlayer region. The radial distribution function diagram between oxygen atoms in water molecules in Fig 16 (c) and the radial distribution function diagram between oxygen atoms and hydrogen atoms in water molecules in Fig 16 (d) reveal the adsorption characteristics of water molecules on the montmorillonite molecule surface. Although the numerical coordinate distributions of the two figures are not completely identical, the shape trends of the functions are basically the same, and the peak values all gradually decrease. This indicates that the stable hydrogen bonds formed between water molecules and the montmorillonite surface are gradually disappearing, and the stability of the montmorillonite molecule is gradually declining. This structural evolution process reveals the intrinsic relationship between the degree of hydration and the stability of the montmorillonite molecule.

### 4.3. Particle transport laws within the interlayer region

**Fig 17:**
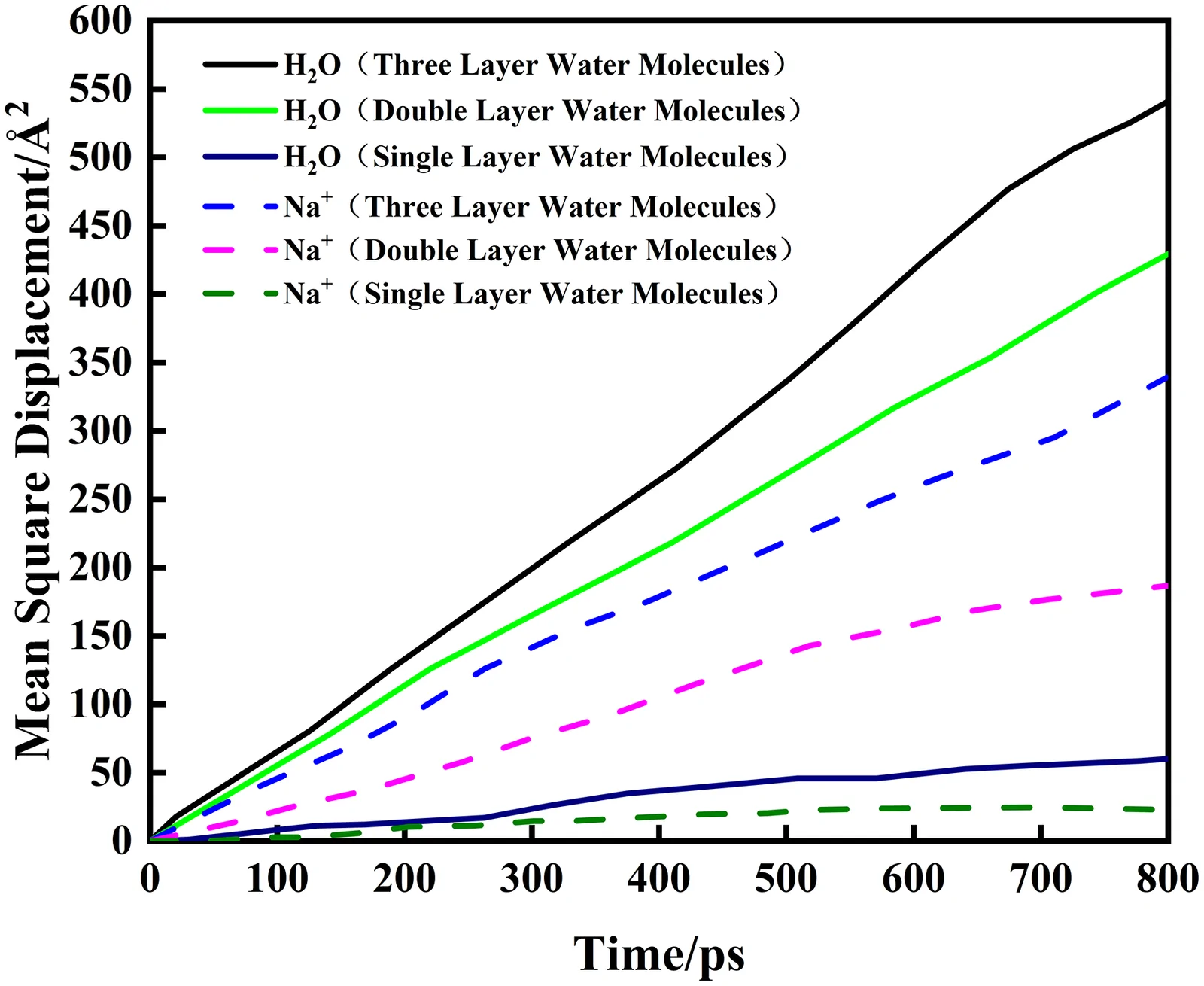
Mean square displacement curve of water molecules and Na^+^ in interlayer region.

**Fig 18:**
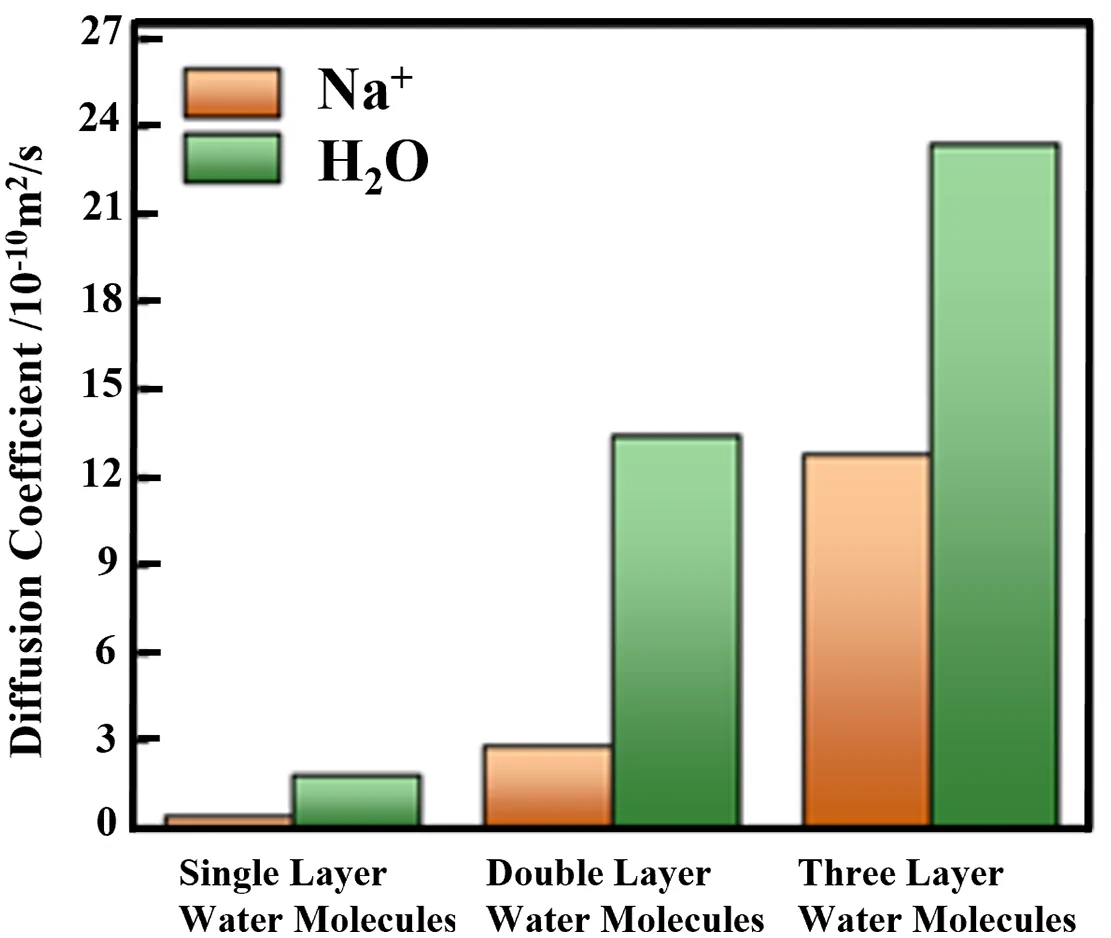
Diffusion coefficient diagram of water molecules and Na^+^ in interlayer region.

According to the principles of statistical mechanics, the transport laws of particles during the hydration process can be revealed. Based on the conductive properties of particles, calculating their mean square displacement curves can obtain the diffusion coefficient, thereby intuitively reflecting the displacement changes of particles over a certain period and used to evaluate the structural stability after the hydration reaction ends. The SPC/E water model is known to overestimate absolute diffusion coefficients to some extent; however, since this study focuses on the comparative trend between water and Na⁺ mobility rather than absolute values, the systematic bias does not affect our qualitative conclusions. Through numerical simulation and formula calculation, the mean square displacement curves and diffusion coefficients of water molecules and Na⁺ were obtained, as shown in Figs 17 and 18.

The 800 ps simulation duration is sufficient for the interlayer water to reach the Fickian diffusion regime, as the MSD curves have already exhibited clear linear behavior within this timescale, consistent with previous MD studies on clay interlayer diffusion. By fitting the linear equation of mean square displacement, the corresponding diffusion coefficients were calculated. It was found that when the interlayer domain contains different numbers of water molecules, the mean square displacements of water molecules and Na⁺ also differ. When a single layer of water molecules forms in the interlayer domain, the diffusion coefficient of water molecules is 1.8 × 10^−10^ m^2^/s, and that of Na^+^ is 0.4 × 10^−10^ m^2^/s. When a double layer of water molecules forms, the diffusion coefficients are 13.4 × 10^−10^ m^2^/s and 2.8 × 10^−10^ m^2^/s for water molecules and Na^+^, respectively. When a three layer forms, they are 23.4 × 10^−10^ m^2^/s and 12.8 × 10^−10^ m^2^/s, respectively. Since the diffusion coefficient of water molecules in the system is greater than that of Na ⁺ , it can be concluded that water molecules have a stronger migration ability and higher mobility within the interlayer domain.

As the water content in the interlayer domain increases, the diffusion coefficients of both water molecules and Na⁺ increase significantly, showing an upward trend. The diffusion coefficient of water molecules is approximately 2–5 times that of Na^+^. This is because the increase in the number of water molecules expands the interlayer domain of montmorillonite molecules, providing channels for the diffusion of water molecules and Na^+^, further promoting their degree of free diffusion. At the same time, it was found that the diffusion coefficient of water molecules is significantly greater than that of Na^+^, because water molecules have better stability compared to Na⁺ and can more effectively exchange with the hydration layer of montmorillonite molecules. Secondly, although the attractive force of the montmorillonite molecular layer on charged particles weakens with increasing water content, it still maintains a certain adsorption capacity for Na^+^. This Coulomb interaction restricts the diffusion of Na^+^, resulting in the diffusion capacity of water molecules being significantly higher than that of Na^+^, leading to different degrees of hydration characteristics in montmorillonite molecules. Under high water content conditions in the interlayer domain, the high diffusivity of water molecules causes the diffusion speed of Na⁺ to increase significantly with the increase in water content. This change seriously disrupts the charge balance of montmorillonite molecules, thereby reducing their structural stability and ultimately leading to expansion instability in the montmorillonite molecular structure.

### 4.4. Parameterized analysis of montmorillonite mechanical properties

With increasing hydration time, a large number of water molecules invade the interlayer region of the montmorillonite molecule, causing a significant increase in interlayer spacing and structural expansion deformation. By tracking the dynamic motion trajectories of system molecules using the Forcite MP module and combining Voigt [26] and Reuss [27,28] theories, a coupling analysis of the number of water molecules, interlayer spacing, and mudstone mechanical parameters was performed. The variation laws of the system’s molecular bulk modulus, shear modulus, elastic modulus, and Poisson’s ratio with increasing water content were obtained, analyzing the changes in mechanical parameters due to hydration.

**Fig 19:**
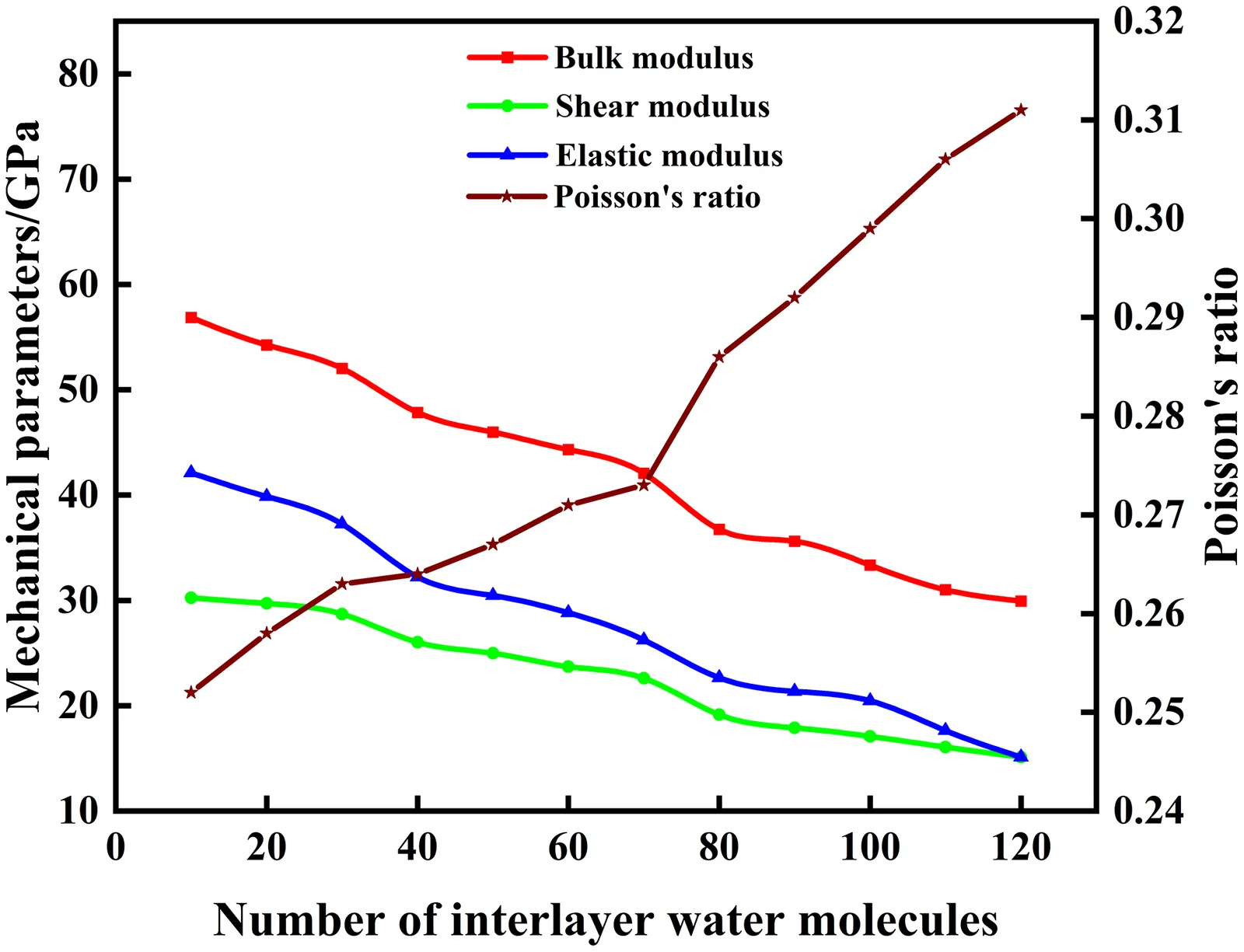
Variation trends of montmorillonite mechanical parameters with the number of interlayer water molecules.

Analysis of the data in Table 5 shows that the effect of hydration on montmorillonite mechanical properties exhibits a systematic, trend-based deterioration pattern. As the number of water molecules increases from 10 to 120, corresponding to interlayer spacing expansion from 10.12 Å to 18.33 Å, the bulk modulus of montmorillonite decreases from 56.85 GPa to 29.93 GPa, a reduction of 47.4%; the shear modulus decreases from 30.25 GPa to 15.12 GPa, a reduction of 50.0%; the elastic modulus sharply decreases from 42.11 GPa to 15.12 GPa, a reduction of up to 64.1%. In stark contrast, the Poisson’s ratio monotonically increases from 0.252 to 0.311, an increase of 23.4%. It should be noted that the moduli calculated here represent the intrinsic mechanical properties of the montmorillonite single crystal; the macroscopic mudstone moduli are additionally influenced by pores, microcracks, and other mineral components. Therefore, the comparison focuses on the consistency of the deterioration trend rather than on absolute numerical equivalence. As shown in Fig 19, when the interlayer spacing exceeds approximately 15 Å (corresponding to about 70 water molecules, i.e., the stable formation of a triple-layer water film structure), the decline rates of all moduli and the increase rate of Poisson’s ratio significantly accelerate. This indicates that the qualitative change of the water film structure (from double-layer to triple-layer) has an accelerating effect on the deterioration of mechanical properties. This evolution law of mudstone mechanical properties revealed from atomic-scale simulations highly coincides with and corroborates the results of the macroscopic mudstone immersion experiments in Section 2.2. In the macroscopic experiments, the elastic modulus of Block S mudstone significantly decreased after immersion in fresh water, and the Poisson’s ratio increased by 20%. The microscopic simulation essentially explains the physical origin of this macroscopic phenomenon: Montmorillonite, as the most critical water-sensitive mineral in mudstone, experiences interlayer expansion, ion hydration, and migration upon contact with water, directly leading to the loss of mineral stiffness and the enhancement of ductility. This “softening” and “ductilization” at the mineral scale manifests macroscopically at the rock scale as a sharp decrease in compressive strength and cohesion, as well as an increase in rock deformation capacity, ultimately inducing the progressive spalling and overall fragmentation of the core as shown in Fig 4. From an engineering perspective, this progressive stiffness degradation implies that the rock strength threshold becomes increasingly sensitive to external drilling loads. Under high‑torque and high‑friction conditions, the weakened montmorillonite crystals are more susceptible to shear failure, and the reduced cohesion lowers the critical stress state required for wellbore collapse. This coupling between water‑induced mechanical deterioration and complex dynamic loading explains why borehole instability often occurs unexpectedly during high‑load operations in water‑sensitive formations.

**Table 5:** Table of rock mechanics parameters.

Number of water molecules	Interlayer spacing (Å)	Bulk modulus (GPa)	Shear modulus (GPa)	Elastic modulus (GPa)	Poisson’s ratio
10	10.12	56.85	30.25	42.11	0.252
20	11.57	54.25	29.71	39.87	0.258
30	12.17	52.03	28.69	37.25	0.263
40	13.03	47.85	26.03	32.23	0.264
50	13.50	45.98	24.99	30.45	0.267
60	14.56	44.32	23.72	28.82	0.271
70	15.63	42.06	22.61	26.24	0.273
80	16.51	36.73	19.15	22.65	0.286
90	17.50	35.62	17.90	21.35	0.292
100	17.99	33.34	17.08	20.47	0.299
110	18.24	31.01	16.06	17.64	0.306
120	18.33	29.93	15.12	15.12	0.311

## 5. Conclusions

Through a multi-scale research method combining macroscopic experiments and molecular dynamics simulations, this paper systematically reveals the macro- and micro-mechanisms of water-induced deterioration in montmorillonite-rich mudstone and its impact on borehole stability. The main conclusions are as follows:

Core mineral analysis shows that montmorillonite is the dominant clay mineral, with an average content of 61.6%. The high montmorillonite content is the fundamental cause of the strong water sensitivity and mechanical deterioration of the mudstone. Immersion experiments confirm that both fresh water and water-based drilling fluid can induce severe deterioration of mechanical properties. After immersion in fresh water, the core’s compressive strength decreases by more than 55%, cohesion decreases by over 51%, accompanied by significant progressive spalling failure.

Molecular dynamics simulation of montmorillonite hydration reveals that the invasion of water molecules into the montmorillonite interlayer is a dynamically developing stage: evolving from a single-layer water film, to a double-layer, and finally forming a triple-layer water film structure, with the interlayer spacing expanding nonlinearly accordingly. Meanwhile, Na⁺ gradually detaches from the crystal lattice, migrates into the interlayer, and forms an alternating structure with water molecules. Its diffusion capacity significantly increases with the intensification of hydration. This microscopic process of “staged hydration expansion” and “ion migration activation” directly corresponds to and explains the macroscopic experimental observation of progressive core failure characterized by “initial spalling followed by fragmentation” with increasing immersion time.

The study establishes a cross-scale correlation for mudstone hydration from “water molecule invasion — interlayer structure evolution — mineral mechanical property degradation — macroscopic rock mass instability,” clarifying the microscopic mechanism of borehole hydration instability in mudstone. Based on the staged water‑film formation and Na⁺ migration mechanisms identified in this study, K^+^ -based inhibitors and polyamine or organosilicon inhibitors are more effective for this type of formation.
